# Delays, interruptions, and losses from prevention of mother-to-child transmission of HIV services during antenatal care in Johannesburg, South Africa: a cohort analysis

**DOI:** 10.1186/s12879-015-0778-2

**Published:** 2015-02-06

**Authors:** Kathryn Schnippel, Constance Mongwenyana, Lawrence C Long, Bruce A Larson

**Affiliations:** Health Economics and Epidemiology Research Office (HE2RO), School of Clinical Medicine, Faculty of Health Sciences, University of the Witwatersrand, Johannesburg, South Africa; Department of Global Health and the Center for Global Health and Development, Boston University School of Public Health, Boston, MA USA

**Keywords:** Patient adherence, HIV prevention, Antiretroviral therapy, Pregnant women, South Africa

## Abstract

**Background:**

Between 2010–2013, South Africa implemented WHO ‘Option A’ for prevention of mother to child transmission (PMTCT), where all HIV-infected pregnant women (from 14 weeks gestation) received zidovudine (AZT) as ARV prophylaxis and initiated CD4 testing at their first antenatal care (ANC) visit. After returning for a second visit to collect CD4 results, women with CD4 counts *≤* 350 were referred to the ART clinic and fast-tracked for initiation on lifelong ART while continuing to visit the ANC clinic every four weeks. Women with CD4 counts >350 were dispensed daily AZT prophylaxis at monthly follow up visits (every 4 weeks). The primary objective of this study was to evaluate adherence of HIV-infected pregnant women to recommended PMTCT services at and after their first antenatal care (ANC) visit.

**Methods:**

We conducted an observational cohort study from August 2012 to February 2013 at two primary health care clinics in Johannesburg, South Africa using routinely collected clinic data from first ANC visit for up to 60 days.

**Results:**

Of the 158 patients newly diagnosed with HIV at their first ANC visit, records indicated that 139 women initiated CD4 testing during their first ANC visit. 52 patients (33% of 158) did not return again to the clinic within 60 days. Of the 118 (84% of 139) women with known gestational age > 13 weeks and known Hb ≥ 8 g/dl who should have received a 4-week supply of daily AZT at first ANC visit, 81 women (69% of 118) had a record of AZT being dispensed. Among the 139 women with CD4 results, 72 (52%) were eligible for lifelong ART (CD4 count ≤350); however, only 2 initiated ART within 30 days.

**Conclusions:**

Loss to initiation of both single and triple ARV therapy, loss to follow-up, and treatment interruptions were common during ANC care for pregnant women with HIV after their first ANC visit.

## Background

Between 2010–2013, South Africa implemented the World Health Organization’s (WHO) ‘Option A’ for prevention of mother to child transmission (PMTCT), where all HIV-infected pregnant women (from 14 weeks gestation) received zidovudine (AZT) as ARV prophylaxis and initiated CD4 testing at their first antenatal care (ANC) visit. Under this policy, after returning for a second visit to collect CD4 results, women with CD4 counts *≤* 350 were referred to the antiretroviral (ART) clinic and fast-tracked for initiation on lifelong ART while continuing to visit the ANC clinic every four weeks. Women with CD4 counts >350 were dispensed daily AZT prophylaxis at monthly follow up visits (every 4 weeks). HIV-negative women were scheduled to visit the ANC clinic every six weeks for a basic package of services including retesting for HIV at 32 weeks gestation. Very little information exists to document implementation of this policy along key steps in the PMTCT cascade. In April 2013, South Africa adopted Option B, where triple therapy through cessation of breastfeeding replaced daily AZT for women not eligible for life-long ART; at the end of 2014, South Africa adopted Option B+, where all HIV-infected pregnant women are eligible for life-long ART regardless of CD4 count. Each new PMTCT policy has expanded access to ART for a larger proportion of pregnant women. However, overall the steps of the PMTCT cascade are the same.

A 2012 evaluation of the PMTCT program in South Africa found that HIV testing during pregnancy was nearly universal (98% uptake) and that the rate of mother-to-child HIV transmission, as measured by 6 week infant testing, had declined to 3.5% [1]. To eliminate mother-to-child transmission in South Africa, implementation of PMTCT services, and adherence along the PMTCT cascade, will be needed. To date, information on these topics remains lacking. Thus, to provide additional information on early adherence to guidelines by patients and providers for ARV treatment or prophylaxis, laboratory testing, initiation, and drug refills, we conducted a prospective and retrospective, observational cohort study of pregnant women newly diagnosed with HIV presenting at two primary health care clinics in Johannesburg, South Africa.

## Methods

This combined prospective and retrospective, observational cohort study was conducted at two Johannesburg public-sector primary health centers based in a semi-urban informal settlement from September 2012 to February 2013. Both sites provided HIV counseling and testing, ANC, PMTCT and ART services as well as other outpatient services to the general population. Women are referred to a 24-hour community health center or hospital nearby for labor and deliveries. Data for this analysis were extracted from routinely collected information included in standardized clinic registers for pregnant women (18 years or older) newly diagnosed with HIV from their first ANC visit for up to 60 days of follow-up. Patients requesting to be transferred to another (non-study) site for further ANC or HIV care were excluded as information about follow-up visits would not be available in the clinic. Patients diagnosed with TB, with known HIV status at first ANC visit, or unknown HIV status at the conclusion of the first visit were excluded from the study as their schedule of clinic visits was different from the standard for newly HIV-infected ANC.

Standard practice at both clinics was for all new ANC patients to attend the clinic on a designated ANC day(s) each week to receive group pre-test counseling for HIV testing from a lay counselor and to have provider-initiated rapid HIV testing. For pregnant women testing HIV-positive, intended practice at the sites was to initiate CD4 testing by having blood drawn and sent to the laboratory and to receive a 4-week supply of AZT prophylaxis (if at least 14 weeks gestation) from the ANC nurse. AZT is counter-indicated for severely anemic women (hemoglobin (Hb) < 8 g/dl) per the South African PMTCT guidelines [2] and thus point of care Hb testing was available at both clinics. No information was available about maternal viral load. Viral load testing is not routinely done in South Africa prior to initiation on ART; guidelines indicate testing after 6 months on treatment.

HIV-infected women were counseled to return in a week to obtain their CD4 test results, at which point they would be referred to the ART section of the clinic on the same premises to be fast-tracked (i.e. within two weeks) for ART initiation if eligible (CD4 ≤ 350). The policy of fast-tracking pregnant women for ART initiation allowed for clinics to start treatment without three sessions for adherence counseling (the standard of care). At the one-week visit to learn CD4 test results, all HIV-infected women were also reminded to return to the clinic in 3 weeks for the next ANC visit and a next 4-week refill of their AZT (if not on ART). Both clinics had also designated one day each week for follow-up ANC visits, for both HIV-infected and uninfected women.

For this analysis, we evaluated adherence to recommended guidelines (intended standard practice) [2] based on 5 primary outcomes representing specific types of visits or steps along the PMCTC cascade within the 60-day follow up period for the study:

1. The proportion presenting for their first ANC visit within 20 weeks gestation;
2. The proportion initiating CD4 testing (i.e. have blood drawn and sent to laboratory) at the first ANC visit;
3. The proportion initiating AZT prophylaxis at their first ANC visit (for all women at least 14 weeks gestation and with Hb ≥ 8 g/dl);
4. The proportion receiving their CD4 test results within 3 weeks (1 week is intended practice);
5. The proportion of known ART-eligible patients initiating ART within 1 month and 2 months from their first ANC visit (within 2 weeks of CD4 test results is intended practice); *OR* returned to the site for AZT refill within 4 weeks of first AZT dispensed for those not eligible for ART.

The study team extracted patient-level data from ANC, laboratory, and clinic registers as well as ART patient files or electronic medical records at the clinics and entered the data into a Census and Survey Processing System (CSPro, US Census Bureau) database. At the time of the study, no individual patient records (medical files) were kept at the clinic for ANC patients. Data were analyzed using STATA (Version 12, College Station, TX). We estimated simple proportions of women meeting the defined criteria for outcomes 1–5 outlined above.

### Ethics

Approval for this study protocol was received from the Institutional Research Board of Boston University Medical Center and from the Human Research Ethics Committee of the University of the Witwatersrand. All prospectively enrolled patients (September 2012 to February 2013) provided informed consent. A waiver of informed consent was granted to retrospectively review clinic records for patients who presented at the clinic in the month of August 2012. All study participants were at least 18 years of age.

## Results

**Figure 1:**
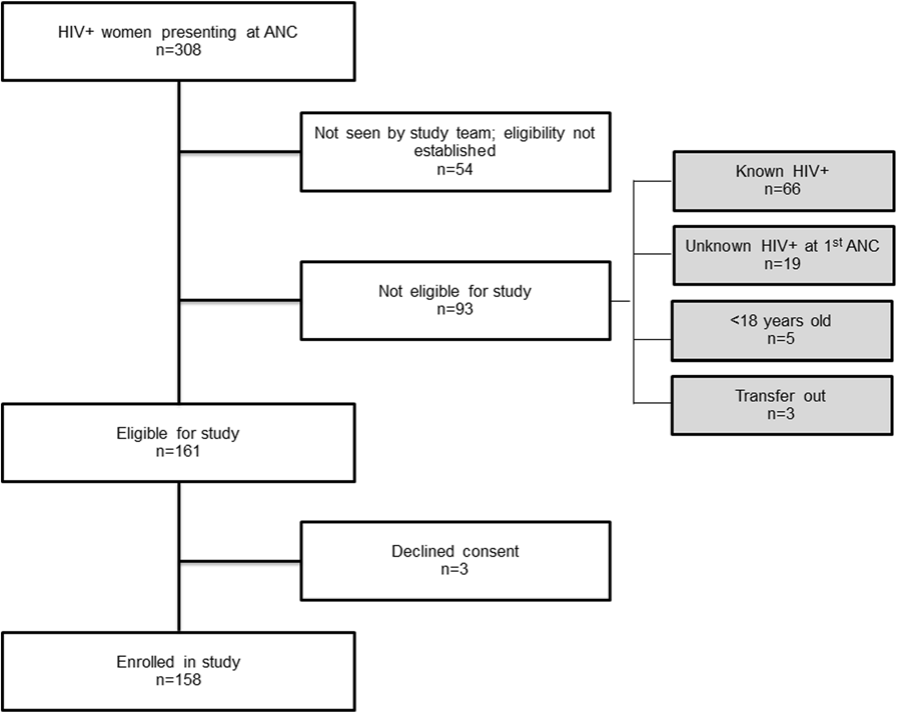
**Patient enrolment and exclusions.**

During the study period, 308 HIV-infected women had their first ANC visit at one of the two clinics. Of these, 54 women received ANC services outside of the designated ANC services days and were therefore not seen by study staff. In addition, 66 pregnant women knew their HIV status prior to the ANC visit during the study period, of whom 25 were already on ART at their first ANC visit. Other exclusions were 3 women who transferred to another ANC site and 5 women who were <18 years of age. 19 HIV-infected women were excluded because of a clinic stock out of HIV rapid test kits; nurses and patients had to wait for laboratory HIV test results to return before treatment could be initiated. Although TB symptom screening was documented in the registers and some women were tested for TB, no women were excluded for TB co-infection. The consort diagram for the study is presented as Figure 1.

Of the 161 HIV-infected pregnant women eligible for the study, 3 declined consent, and 107 and 51 were enrolled at each clinic, for a total of 158. Women presented at a median age of 26 years (IQR: 24 – 30) and during their 2nd pregnancy. Patient characteristics are presented in Table 1.

**Table 1:** **Patient characteristics, stratified by ART eligibility**

	**All women**	**CD4 ≤ 350**	**CD4 > 350**
Enrolled in study	158	72	67
Maternal age, years	26 (IQR: 24–30)	27 (IQR: 25–31)	23 (IQR: 25–29)
Gravidity	2 (IQR: 2–3)	2 (IQR: 2–3)	2 (IQR: 2–3)
Parity	1 (IQR: 1–2)	1 (IQR: 1–2)	1 (IQR: 1–2)
Median gestational age at first ANC, weeks	24 (IQR: 19–28)	24 (IQR: 20–29)	23 (IQR: 18–27)
Median CD4 count	340 (IQR: 197–468)	211.5 (IQR: 134–262.5)	472 (IQR: 410–651)

Table 2 presents results for each primary outcome, stratified by ART eligibility. We summarize the key results for each primary outcome below.

**Table 2:** **Adherence to PMTCT guidelines, stratified by ART eligibility**

	**All women**	**CD4 ≤ 350**	**CD4 > 350**
	**158**	**72**	**67**
*1. First ANC visit prior to 20 weeks gestation*			
No record of gestational age, last menstrual period, or expected date of delivery	8 (5%)	4 (6%)	4 (6%)
<20 weeks gestation at first ANC	39 (26%)	15 (21%)	20 (30%)
*2. Initiate CD4 testing at first ANC visit*			
CD4 tests results from first ANC found	139 (88%)	72	67
WHO clinical stage found	86 (54%)	43 (60%)	32 (48%)
*3. Initiate AZT prophylaxis when first eligible (at least 14 weeks gestation and Hb ≥ 8 g/dl)*			
>13 weeks gestation at first visit	141 (89%)	65 (90%)	58 (87%)
Hb ≥ 8 g/dl at first visit	132 (84%)	56 (78%)	60 (90%)
Known eligible for AZT	118 (75%)	51 (72%)	52 (78%)
Received AZT at first visit, if eligible	81 (69%)	37 (73%)	32 (62%)
*4. Receive CD4 count results*			
Have CD4 results visit recorded	53 (38%)	36 (50%)	17 (25%)
Made a return visit to clinic <3 weeks	38 (24%)	24 (33%)	13 (19%)
Made a return visit to within 60 days	96 (69%)	47 (65%)	49 (73%)
Median days to CD4 results received (n = 106)	28 (IQR: 8–34)	19 (IQR: 8–29)	29 (IQR: 8–36)
*5. Initiate ART within 30 days of first ANC or receive 60-day uninterrupted supply of AZT*			
Initiate ART within 30 days of first ANC	N/A^/1^	2 (3%)	N/A
Initiated ART within 60 days of first ANC	N/A	15 (21%)	N/A
Median days to ART initiation (n = 15)	N/A	47 (IQR: 37–57)	N/A
Weeks gestation at ART initiation (n = 15)	N/A	27 (IQR: 24–33)	N/A
Receive 60-day uninterrupted supply of AZT	N/A	N/A	10 (15%)
Received 60-day supply of AZT within 60 days	N/A	N/A	20 (30%)
Median days from first AZT to 2nd AZT (n = 20)	N/A	N/A	31 (IQR: 29–36)

/1 N/A = not applicable.

### Outcome 1. Presented for first ANC prior to 20 weeks gestation

Gestational age could not be determined for eight women, meaning that last menstrual period, gestational age, and expected due date were all missing from clinic records. The median gestational age was 24 weeks (IQR: 19–28) at their first ANC visit; 39 (26% of 150) of women attended ANC prior to 20 weeks gestation, and only nine women (6% of 150) presented at first ANC prior to 14 weeks gestation.

### Outcome 2. Initiated CD4 testing at first ANC visit

19 women did not have records of initiating CD4 testing at their first ANC visit. For 139 who initiated CD4 testing at their first visit, and thus have a CD4 test result, the median CD4 count was 340 (IQR: 197–468). 72 (46%) were eligible for lifelong ART (triple therapy) according to guidelines in place at the time (CD4 count ≤350) at their first ANC visit. Clinical staging was poorly documented; 86 (54%) of women had a WHO clinical HIV stage recorded. Of these, only 1 woman was recorded as stage 3 and therefore eligible for ART regardless of CD4.

### Outcome 3. Initiated AZT prophylaxis at first ANC visit for eligible women

No Hb result was recorded for 18 women (11%). Of the women with pregnancies at least 14 weeks gestation (n = 141) at their first ANC visit, 7 had an Hb level that was less than 8 g/dl. Of the 118 (84%) women with known gestational age at least 14 weeks and known Hb ≥ 8 g/dl who should have received a 4-week supply of daily AZT at their first ANC visit, 81 women (69%) had a record of AZT being dispensed at their first ANC.

### Outcome 4. Received CD4 count results within 3 weeks of first ANC visit

52 patients (33% of 158) did not return to the clinic within 60 days of their first ANC visit. For the 67% of women who did return at least once within 60 days of their first ANC visit, 30% returned within 3 weeks of the first ANC visit. For those who returned at least once during the 60-day follow up period, the median number of days from their first to second visit was 28 (IQR: 8 – 34).

### Outcome 5a. For those known to be eligible for ART, initiated ART within 30 days of their first ANC visit

ART records indicated that 2 out of 72 women known to be eligible for lifelong ART (3%) initiated ART within 30 days of their first ANC visit (i.e. fast-tracking worked), and a total of 15 of 72 women (21%) initiated ART within 60 days. The median number of days from first ANC visit until ART initiation was 47, meaning that women were starting ART only at the beginning of their third trimester for those who initiated treatment at the site.

### Outcome 5b. For those not eligible for ART, received an uninterrupted supply of AZT during the 60-day follow up

According to ANC registers, only 10 out of 67 women with CD4 > 350 (15%) received an uninterrupted 60-day supply of AZT; i.e. only 15% of women returned on time for their AZT refill after 4 weeks. Among those not returning on time, 18 (32%) did not return again during the 60-day follow up period.

## Discussion

In this combined prospective and retrospective observational cohort study, clinic records for HIV-infected pregnant women indicated multiple delays and interruptions as well as loss to follow-up in the initiation and continuation of AZT and lifelong ART. Maternal age, gravidity, parity, and baseline CD4 count of ART-eligible women diagnosed with HIV at first ANC were similar to previously reported studies in South Africa; median gestational age was slightly younger [3-5]. In this study, ART-eligible women presented at a median gestation age of 24 weeks. The proportion of ART-eligible women who initiated treatment within 60 days was surprisingly low (21%). While women who did not initiate ART within the 60 days follow-up may still have initiated ART prior to delivery, duration on ART, and therefore the potential effectiveness of ART [6,7], would have been limited. The long delays in initiation, treatment interruptions, and high loss to treatment found in this study occurred despite large-scale implementation of interventions in PMTCT programs in South Africa, including fast-tracking of pregnant women with reduced adherence counseling and ART preparation, nurse-initiation of ART, and integration of ANC and ART services at the same clinic.

Initiation of AZT was also below expectation. According to clinic files, only 69% of women who were documented as being eligible for initiation of AZT received AZT at their first visit. 11% of women were missing Hb test results, which has clinical implications for the management of anemia as well as AZT eligibility. Problems which limited the same day initiation of AZT in this study are likely to carry over to the new Option B+ guidelines implemented in South Africa, which call for all HIV-infected women to receive a fixed-dose, triple therapy combination at first ANC visit.

The current study was not designed to determine the cause of the problems with initiation and adherence. Clinic managers and nursing staff at the sites indicated that AZT, when available, was always dispensed. However, multiple health systems issues that have been identified elsewhere as barriers to adherence to PMTCT regimens [8] were encountered during the study period. One clinic experienced a stock-out of HIV rapid test kits and enrollment was interrupted as women had to return to the clinic for laboratory-generated HIV results before initiating CD4 test and receiving AZT. The other clinic experienced repeated stock-outs of AZT, including one period of more than 6 weeks (just prior to the study period) when AZT was not available. Health care worker shortages caused difficulties for patient care as well, especially during holiday periods as staff members on leave for studying, vacation, or illness were not replaced. Thus, women may have presented for care but been turned away because of staff, drugs, or consumable shortages.

The two clinics were selected for convenience reasons and may not be generalizable to other clinics within South Africa. However, both clinics are located within the city of Johannesburg and thus, on average, are more likely to have access to staffing, consumables, and supervision than facilities in more rural and remote areas.

The study was designed to evaluate the early steps of the PMTCT cascade, and in particular whether interventions such as same day initiation of AZT for all women and fast tracking of ART initiation for eligible women were occurring; therefore, a short period of follow-up was chosen. This study also did not match mother-infant pairs to determine if the delays, losses and interruptions documented here affected transmission; it may be that treatment is robust despite these challenges. While women may have initiated ART, continued AZT beyond the 60 days, or delivered a healthy, HIV-free baby, earlier initiation maximizes the opportunity for prevention [6,7].

A limitation of this study is that it was a review of clinic records; it may be that clinical practice differed from what was recorded in clinic records. However, for HIV-infected persons initiating ART, patient medical information is written in individual patient files, kept by the facility and stored within the ART clinic; one of the clinics also captured patient files into an electronic medical record system for HIV management. For this set of pregnant women, who are both ANC and ART patients, their medical records from first presentation at the ART clinic within the sites are as good as for any other ART patients (and only 21% known eligible initiated treatment with 60 days of HIV testing).

However, within the ANC clinic itself, no individual clinic-maintained patient files or clinic cards were in use at the time of the study. ANC patient cards are to be completed at each visit and women are strongly encouraged to bring these patient cards to each visit and at delivery. Nursing staff are asked to duplicate, by hand, information recorded in the patient cards in the clinic ANC register as well. It may be that some of the missing visits identified in this analysis were completed by the patients and recorded in the patient cards but never recorded in the ANC clinic register.

Each ANC clinic had one ANC register, which is a very large format hardcover, pre-printed book with follow-up visits marked at 6-weekly intervals. Because each clinic had one large register, it was kept in a central location. With more than one nurse providing antenatal care, so that the single ANC register could not always be in front of the nurse for initial or follow-up visits, nurses often could not enter patient information into the clinic’s ANC register during the consultation. Nurses solved this constraint by writing patient-visit information onto separate sheets of paper during a patient interaction, with the intent of later transcribing such information back into the ANC register. This may have been a reason for poor capturing into the register of Hb, gestational age, and WHO staging. During the study period, however, study staff remained at the sites to complete this transcribing for the sites so that information extracted from the ANC register during the study period was as complete as information obtained at the sites. Study staff also used a pregnancy due date wheel tool from the clinic to fill in missing information for last menstrual period, weeks gestation at time of clinic visit, or expected delivery date. Beyond the study period, such after-the-fact transcribing into the large-format ANC register was no longer completed by study staff and remains a barrier to information management at the sites.

Printed registers and patient cards with follow-up visits marked at 6-weekly intervals for ANC care of HIV-negative women may also have contributed to poor adherence by both the facilities and the patients to the increased number of visits required for ANC care if HIV-infected. Patients were told during their first visit to return in a week for CD4 results, dispensed a 4-week supply of AZT, and received an ANC patient card indicating 6-week visit. For those who returned for a second for a second visit, the median time from first visit to CD4 results was 28 days, coinciding with a need to return to the clinic for a 4-week AZT refill.

Additional registers (e.g. recording return visits for CD4 results, referral to ART initiation, and counseling for ART initiation) were in use throughout the two clinics, mostly informal hand-drawn columns in notebooks. These registers were organized according to the particular use of that section of the clinic (e.g. date of first visit, date of current visit, patient name, patient date of birth, or ANC register number), which caused difficulties in tracing patients through the services. Neither clinic site had dedicated staff for assisting in record keeping or data management for these patients; therefore, study staff assisted the clinics in collecting information from the different sources and completing registers during the period. Again, beyond the study period, the time required for this after-the-fact tracing of patients across the multiple registers is a barrier to information and patient management at the sites.

## Conclusions

This review of clinic records on initiation of AZT prophylaxis and ART for newly-identified HIV-infected women presenting for ANC care in Johannesburg found multiple delays and gaps in scheduled visits, delays in initiation of appropriate treatment, and gaps in treatment once initiated. Consolidation of registers and adaptation of clinic records to current guidelines, so that it is possible for clinics to capture information completely and accurately, in a manner that is feasible for clinic staff to complete, remains an important first step to identifying the cause of the delays, interruptions and loss to care documented in this study.
